# Functional, Chemical, and Phytotoxic Characteristics of *Cestrum parqui* L’Herit: An Overview

**DOI:** 10.3390/plants13152044

**Published:** 2024-07-25

**Authors:** Maria Chiara Di Meo, Cinzia Di Marino, Pasquale Napoletano, Anna De Marco, Anna Rita Bianchi, Silvana Pedatella, Domenico Palatucci

**Affiliations:** 1 Department of Sciences and Technologies, University of Sannio, 82100 Benevento, Italy; mardimeo@unisannio.it; 2 Department of Chemical Sciences, University of Naples Federico II, 80126 Naples, Italy; cdimarin@unina.it (C.D.M.); silvana.pedatella@unina.it (S.P.); 3 Department of Agriculture, Food, Environment and Forestry (DAGRI), University of Florence, 50144 Florence, Italy; 4 Department of Pharmacy, University of Naples Federico II, 80131 Naples, Italy; ademarco@unina.it; 5 Department of Biology, University of Naples Federico II, 80126 Naples, Italy; annarita.bianchi@unina.it

**Keywords:** *Cestrum parqui* L’Herit., insecticidal and antifeedant activity, herbicidal activity, secondary metabolites, lignans, flavones, oxylipins

## Abstract

*Cestrum parqui* L’Herit. (Solanaceae family) is a species of forest shrub, self-incompatible and specialized in pollination, widespread in the subtropical area of the planet, and now widely distributed also in the Mediterranean area. The constituents of its leaves have antimicrobial, anticancer, insecticidal, antifeedant, molluscicidal, and herbicidal properties. The spread of this species represents a valuable source of compounds with high biological value. Various research groups are engaged in defining the chemical composition of the different parts of the plant and in defining its properties in view of important and promising commercial applications. To date, there are only a few incomplete reports on the potential applications of *C. parqui* extracts as selective natural pesticides and on their potential phytotoxic role. Scientific knowledge and the use of extraction techniques for these components are essential for commercial applications. This article summarizes the research and recent studies available on the botany, phytochemistry, functional properties, and commercial applications of *C. parqui*.

## 1. Introduction

**Figure 1:**
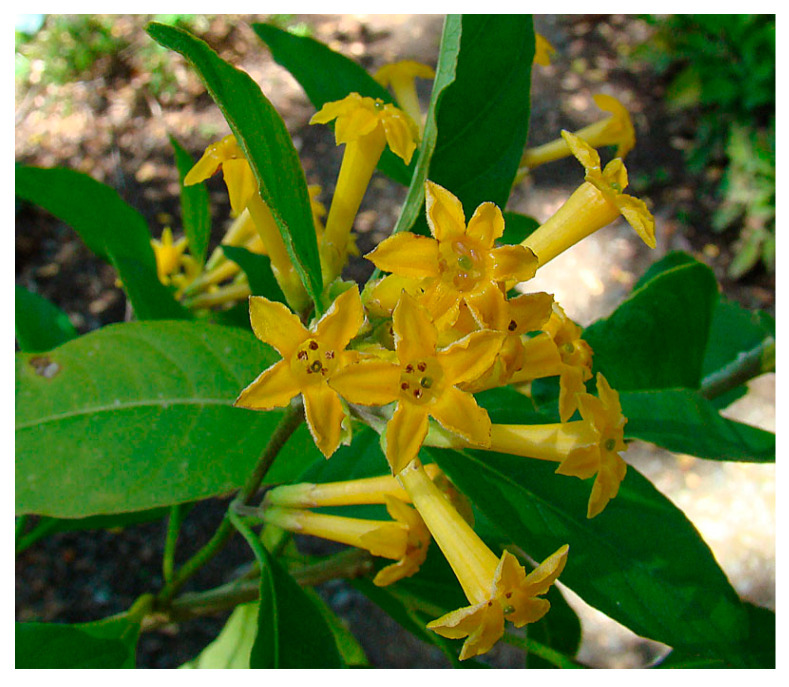
Flowers of *Cestrum parqui* L’Herit.

Plants synthesize numerous secondary metabolites through specific metabolic pathways [1]. These molecules are interesting for plant ecology, reproduction, and physiology. The classes and types of secondary metabolites produced are a useful tool in phylogenetic and taxonomic studies [2]. Humans have exploited natural substances for a long time in medicinals, agriculture, arts, food, feed, and religion. Specifically, the application of natural products in agriculture is nowadays leveraged in order to reduce human’s cultures impact on ecosystems and on public health [3]. This review is focused on *Cestrum parqui* L’Herit’s (green cestrum, Figure 1) secondary metabolites content and the effects of plant extracts and purified chemicals in pest and weeds controlling. This plant belongs to the Solanaceae family and has been cultivated throughout the world in gardens as an ornamental species. *Cestrum parqui* is also known as Chilean cestrum, Chilean flowering jessamine, Chilean jessamine, green cestrum, green poison berry, green poison-berry, green poisonberry, iodine bush, willow jasmine, willow leaved jessamine, willow-leaf jessamine, willow-leaved jasmine, and willow-leaved jessamine [4]. It was been first described by Carolus Ludovicus L’Heritier in 1785 [5], and its origins trace back to South America [6]. Nowadays, it is widely diffused all over the world, and classified according to the new systematic [7,8].

*Taxonomy, Morphology, and Distribution*

**Figure 2:**
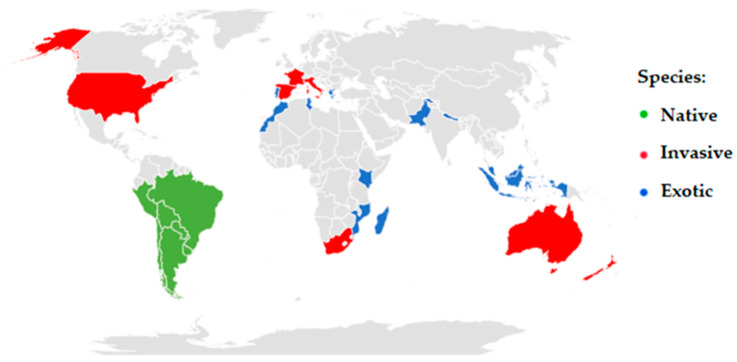
World distribution of *C. parqui*.

*C. parqui* belongs to the Solanaceae family, a family of dicotyledonous angiosperms largely cultivated by humans as horticultural—*Solanum tuberosum*, *Solanum melongena*, *Solanum lycopersicum*, and *Capsicum annuum*, medicinal—*Atropa belladonna*, and recreational crops—*Nicotiana tabacum*. The family comprises 100 genera with about 2500 species. The genus that hosts the largest number of plants is *Solanum*, with about 1400 species; conversely, *Licianthes* includes 200 species, and *Cestrum* has 175 species [9,10]. Solanaceae are represented in the wild on all continents, with a greater number of species in the American continent, and they adapt well to almost all ecosystems, although most of them prefer warmth rather than intense cold. *C. parqui* is native to Brazil, Bolivia, northern and central Chile, Peru, Paraguay, Uruguay, and northern and central Argentina, but, today, it is widely spread in southeastern and eastern parts of Australia, New Zealand, in some parts of the southern United States of America like California and Texas, and in much of Europe (Figure 2) [11].

This plant is one of the main weeds of the Mediterranean area [12]. Its spread began as an ornamental plant, but it soon became invasive in many warm temperate and subtropical regions because it adapts well to the edges of watercourses and is also found in parks, old gardens, uncultivated areas, open woods, forest edges, pastures, and along roadsides. In Australia, *C. parqui* is considered an environmental weed, meaning it has no agricultural function, damaging and competing with existing plants, especially in New South Wales and Queensland [13]. For this reason, it is currently listed as a priority environmental weed in three regions and a sleeper weed in other parts of the country [14]. The invasiveness of this plant is particularly evident when it forms dense stands along forest edges and watercourses, replacing native plants in these habitats and preventing their regeneration [13]. It is an erect, highly branched shrub that usually grows 1–3 m tall, but occasionally reaches up to 5 m in height. It has tubular flowers in clusters, yellow or greenish-yellow in color, and stems and leaves that have an unpleasant odor when crushed. The taxonomy of the plant is described in Table 1.

## 2. Traditional Use and Properties

In Chilean folk medicine, it was used as an antipyretic and for the treatment of fever and inflammation [15,16]. Extracts of the plant obtained with solvents of different polarity have shown moderate antimicrobial activity against the fungi *Penicillium expansum* and *Candida albicans* and the bacteria *Bacillus subtilis*, *Pseudomonas aeruginosa*, *Staphylococcus aureus*, *Escherichia coli*, and *Streptococcus pneumoniae* [17]. The methanolic extract of the leaves has exhibited possible anticancer activity on the human myeloid leukemia cell line (HL-60) and an antiproliferative effect on another two cell lines (HT-29 and Molt-3 cells); this may be due to the presence of ursolic and oleanolic acids, two pentacyclic triterpenes [18,19], as well as the ability to inhibit platelet aggregation induced by ADP and/or collagen, both in sheep and human blood [20]. Furthermore, the methanolic extract of *C. parqui* leaves has a strong effect on sperm motility in vitro. Electron microscopy studies on human sperm, incubated with concentrations ranging from 40 to 250 µg/mL of *C. parqui* leaf extract and at time intervals ranging from 5 to 240 min, have shown damage to the head and acrosomal membranes, with a maximum spermicidal effect at the highest tested concentration, generally dose- and time-dependent [21]. A screening among *Cestrum* spp. reveals *Aspergillus terreus* as an endophytic fungus of *C. parqui* leaves. This microbe biosynthesizes camptothecin, a modified monoterpene indole alkaloid used in cancer chemotherapy [22,23].

## 3. Potential Effects of *C. parqui*

### 3.1. Insecticidal and Antifeedant Activity 

The genus *Cestrum* is rich in saponins, and most species exhibit toxicity that supports their use as potential insecticides, herbicides, molluscicides, antimicrobial agents, and antitumor agents. In fact, as far back as the early 1950s, the discovery of gitogenin and digitogenin in the green berries of *C. parqui* [24], or tigogenin and digallogenin in dried leaves, has been documented [25].

The leaves of *C. parqui* are the most studied organ of the plant. This is likely related to the observation that many animals that had eaten its leaves were severely intoxicated. For example, several cases of cattle poisoning occurred in Chile between 1992 and 1998 and in Brazil starting from the late 1960s [26]. Necropsies of the animals showed pulmonary edema, congestion, hemorrhages in various organs, and hepatic dysfunction [27]. A few years later, Babouche et al. [28] demonstrated that the saponin-rich fraction obtained from the hydroalcoholic extract of the plant could interfere with insect metabolism by lowering the amount of cholesterol needed for ecdysone production, a molting hormone [29].

*C. parqui*’s insecticidal activity has been tested on different species. Specifically, aqueous extracts of *C. parqui* have been evaluated on *Ceratitis capitata*, commonly known as the Mediterranean fruit fly, at different concentrations [30]. *C. capitata*, widespread in Africa, the Mediterranean basin, and South America, is a highly polyphagous species whose larvae develop in a wide range of fruits and are responsible for significant economic damage to the agricultural sector [31]. Water and organic solvent plant extracts have been tested. The aqueous extract at 0.6% (*w*/*w*) of the plant completely inhibited the pupation process of the neonate larvae, while extracts obtained with organic solvents were almost harmless. In another experiment, ethanolic and water extracts obtained from young and old leaves were tested on *Xanthogaleruca luteola* adult insects. In this case, higher mortality has been obtained for ethanolic extract of young leaves [32]. The leaves are likewise active against desert locust *Schistocerca gregaria*. Some of the contained chemicals interfere with the exuviation process, causing insect death at different evolutionary stages [33].

These results were confirmed a few years later by Chaieb et al. [34,35]. The authors evaluated the entomotoxic activity on *Schistocerca gregaria*, a polyphagous and voracious grasshopper that feeds on leaves, flowers, shoots, fruits, and seeds of various plant species, including numerous species of primary importance to humans such as rice, barley, corn, sorghum, sugarcane, cotton, date palm, and banana; *Spodoptera littoralis*, an insect that can attack numerous economically important crops such as turnips, tomatoes, hemp, hibiscus, purslane, mint, clover, tobacco, mallow, apple, grapevine, and many others; *Tribolium confusum*, an insect that mainly feeds on natural products such as cereals and flour, rice, dried fruit, powdered milk, mouse baits, spices, and corn; and *Culex pipiens*, the most common mosquito in the Northern Hemisphere, hematophagous and harmful to health. Chaieb and colleagues [34,35] performed toxicity tests based on the species, through simple contact, injection, forced ingestion, or addition to the food substrate. In the case of contact tests, the results were modest, probably because the saponins were unable to penetrate the waxy cuticle of the target organisms, evidently due to their hydrophilicity; while in ingestion tests, the food substrate was probably unpalatable to the target animal. The best results were obtained with injection, which is obviously impractical in daily practice. However, the results show greater activity on *S. littoralis* followed by *C. pipiens*, and slightly less on *S. gregaria* and *T. confusum*. In any case, the chances of using the crude material as it is, to be added to the diet of the target organism, seem slim. Apparently, the added product had lost its palatability, suggesting the need to isolate the saponins present in the crude material for individual use. It is not disregarded that the problem can be overcome by delivering the saponins through softer and more palatable foods preferred by the target insects.

The antifeedant effect of the aqueous extract of *C. parqui* has also been measured on *Pieris brassicae*, a butterfly that mainly feeds on cultivated varieties of Brassicaceae, especially *Brassica oleracea* (cabbage), and plants of the genus *Tropaeolum* [36]. The effect of increasing amounts of extract, added in percentages of 2, 4, 8, 16, and 32% to the lepidopteran’s diet, was measured, showing a delay in larval growth at lower concentrations, abnormal metamorphosis at intermediate concentrations, and death at the highest concentration.

It is interesting to note that it has been proven that the activity significantly decreases with the loss of the sugar bound to the steroid nucleus [37], much like what happens in the case of α-chaconine and α-solanine. Thus, it is not surprising that the saponins of *C. parqui* are completely ineffective against the phytopathogenic fungi *Fusarium solani* and *Botrytis cinerea*, which probably can secrete detoxifying enzymes capable of hydrolyzing the sugar chains [38].

For economic and especially environmental reasons (reassuring an increasingly reluctant civil society to the use of chemical products), many research groups are committed to identifying specific natural insecticides. For example, pine wood is particularly susceptible to colonization by organisms of the genus *Leptographium* spp. *Ophiostoma* and *Ceratocystis*, which, by invading the vessels, block the passage of sap and cause deterioration phenomena, including the death of plants. The activity of these pathogens, which is only visible by stripping the trunks of dead or suffering plants, is preceded by that of their vectors, mostly coleopteran insects like *Hylurgus ligniperda*, which, by invading plants weakened by various stresses, let the fungus penetrate the sapwood, following their galleries. The symptoms of the infection consist of a wilting of the canopy, with needles quickly turning from pale green to brown and drying up. Wood assortments undergo considerable depreciation due to aesthetic defect. To date, *H. ligniperda* is controlled using methyl iodide, a chemical product dangerous for users and the environment. Huanquilef et al. [39] tested various fractions of the ethanolic extract of *C. parqui* leaves on *H. ligniperda*. Thus, the fractions soluble in chloroform, ethyl acetate, and butanol, which were then added to the insect’s diet, were considered. Over the course of seven days, it was demonstrated that the considered extracts influenced the feeding behavior of the target organism in both adult organisms and larvae, with a dose-dependent effect. In particular, the chloroform extract was the most active, even at low concentrations (0.4% *w*/*v*), indicating that it can be considered for an economical and relatively simple commercial application. 

### 3.2. Molluscicidal Activity 

In the past twenty years, several authors who have tested the insecticidal and antifeeding activity of hydroalcoholic extracts from leaves have also tested their molluscicidal activity, using the snail *Theba pisana* as a target organism [34,35,40], a gastropod mollusk introduced into numerous areas including northern Europe, North America, parts of Africa, Asia, and Australia, where it has often become an invasive species, posing a serious problem for agriculture. These extracts are mostly produced from leaves dried at 40 °C for four days and then finely powdered. They are first extracted with petroleum ether to remove fats and then with methanol. The methanolic extract is then washed with diethyl ether, causing the precipitation of the saponin-containing fraction, which is used for various types of experiments. In some experiments, the reaction mixture was deposited at the bottom of containers where the snails were free to move, or the mixture was applied directly to the bodies of the target organisms. These snails responded to the presence of the saponin-containing mixture with a strong production of mucus, which caused their dehydration and, ultimately, death. In other experiments, the saponin-containing mixture was added to corn bran or deposited on cabbage leaves, which the snails normally feed on, or was dissolved in water in varying amounts from 2 to 8 mg/mL. In these latter three cases, the effects were minimal and mostly reduced to a slight weight loss in the animals, which stopped eating or drinking, evidently recognizing the presence of the toxic substance [40]. However, it is likely that administering the saponin mixture in different and more palatable foods could yield better results than simple contact. Considering the good results obtained, experimentation on *T. pisana* continued with the aim of understanding if there were differences in the toxicity of the saponin-rich crude material on the juvenile or adult form of the mollusk [41]. Two tests lasting 24 h each were used, and each was repeated three times. In the first test, the saponin-containing fraction was deposited on the surface where the snails moved at concentrations of 10, 100, 500, 1000, and 2000 ppm, respectively. A mortality rate of 100% was found with a concentration of the analyzed fraction equal to 315 µg/cm^2^ for adults and 157 µg/cm^2^ for juveniles, with LD_50_ values of 36 and 6 ppm, respectively. In the second test, the saponin-containing fraction was placed in direct contact with the back of the target organism in quantities of 1, 5, and 10 mg, respectively. A mortality rate of 100% was found with a crude quantity of 10 mg for adults and 5 mg for juveniles, with LD_50_ values of 2.6 and 1.0 mg/animal, respectively.

### 3.3. Phytochemical Composition

**Scheme 1:**
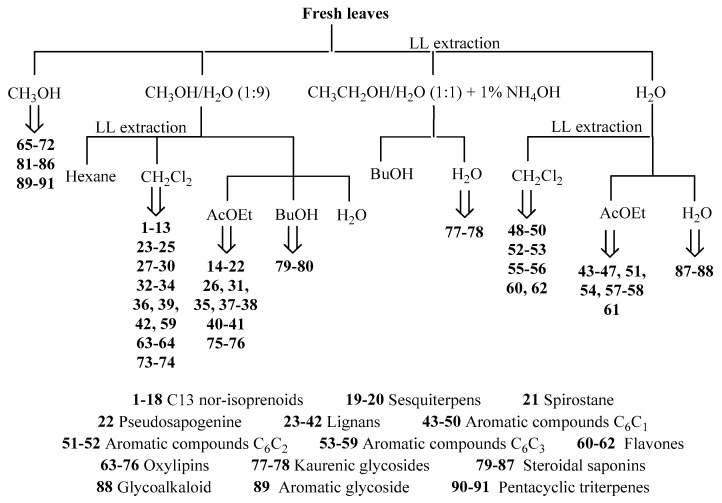
Isolation of C_13_-nor-isoprenoids, sesquiterpenes, spirostanes, pseudosapogenins, lignans, aromatic compounds, flavones, and oxylipins.

**Figure 3:**
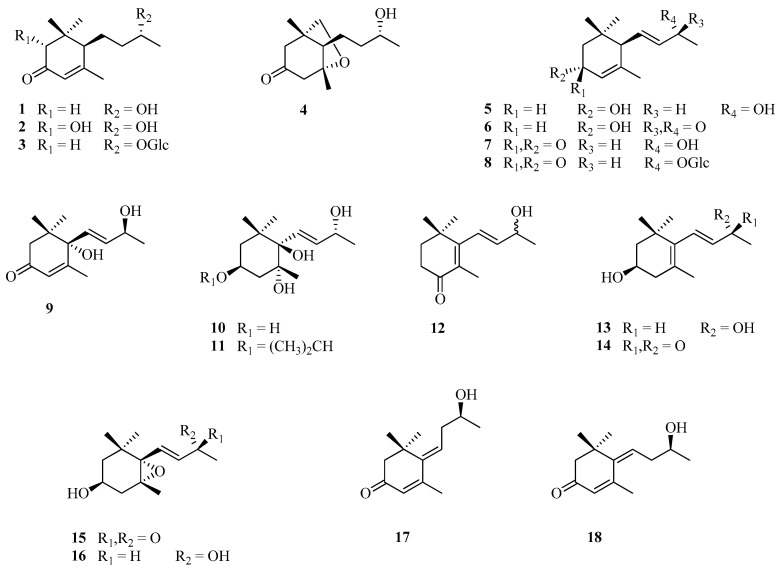
C_13_-nor-isoprenoids isolated from *C. parqui*.

**Figure 4:**
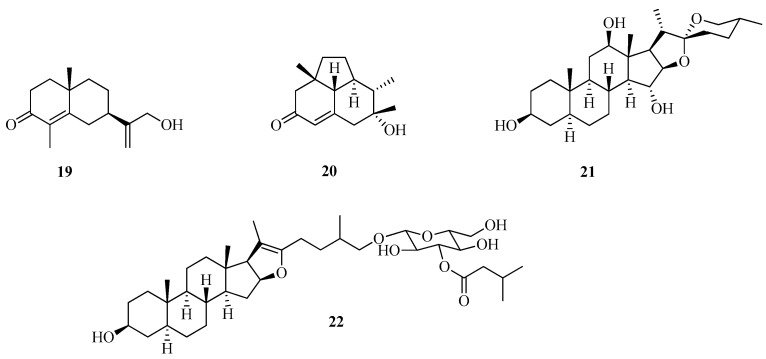
Sesquiterpenes (**19**–**20**), spirostane (**21**), and pseudosapogenin (**22**) isolated from *C. parqui*.

**Figure 5:**
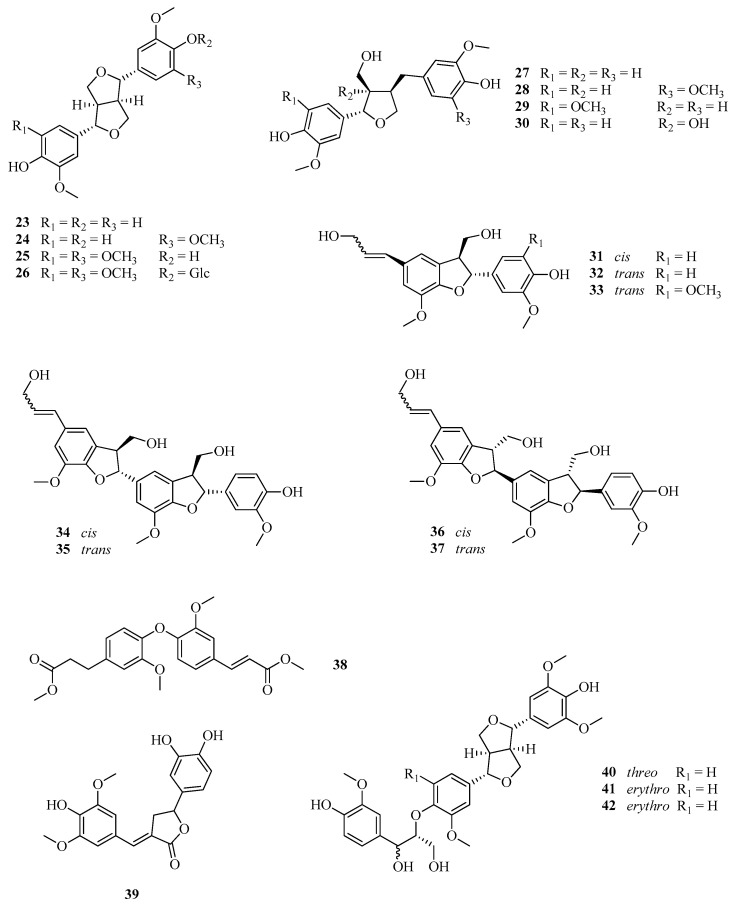
Lignans from *C. parqui*.

**Figure 6:**
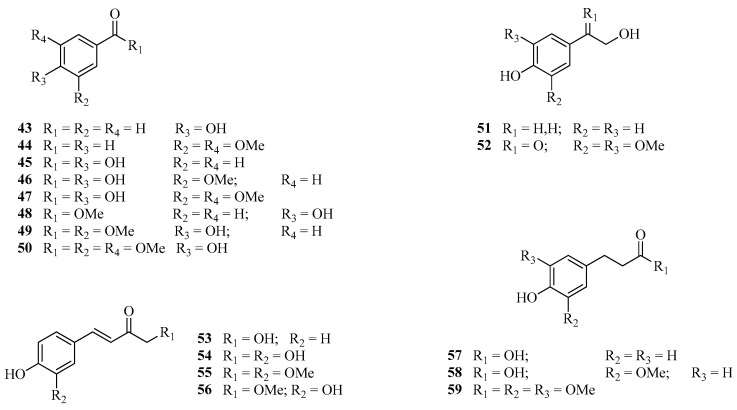
Aromatic compounds C_6_C_1_ (**43**–**50**), C_6_C_2_ (**51**–**52**), and C_6_C_3_ (**53**–**59**) from *C. parqui*.

**Figure 7:**
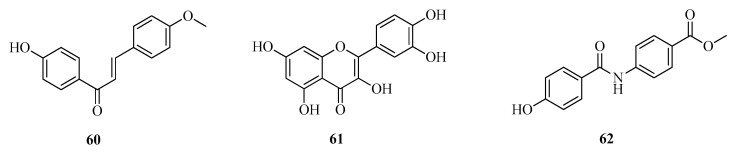
Flavonoids from *C. parqui*.

**Figure 8:**
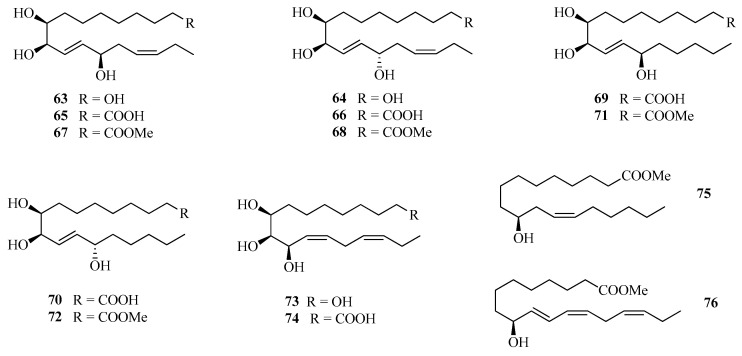
Polyol fatty acids **63**–**64** and **73**, methylated oxylipins **67** and **68**, and oxylipins **65**–**66**, **69**–**72**, and **74**–**76** (Note that **63**/**64**, **65**/**66**, **67**/**68**, **69**/**70**, and **71**/**72** are five diastereomeric couples of compounds).

**Figure 9:**
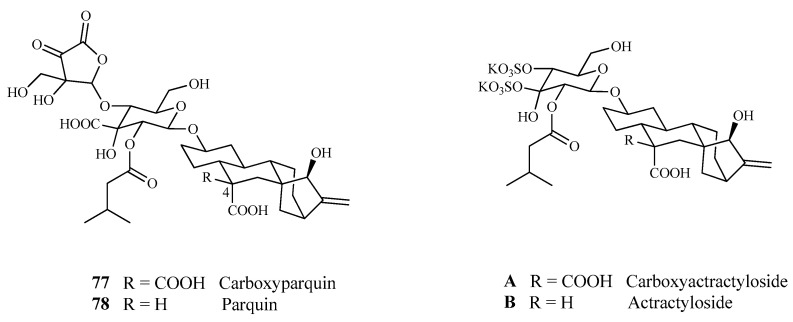
Kaurenic glycosides (**77**–**78**).

**Figure 10:**
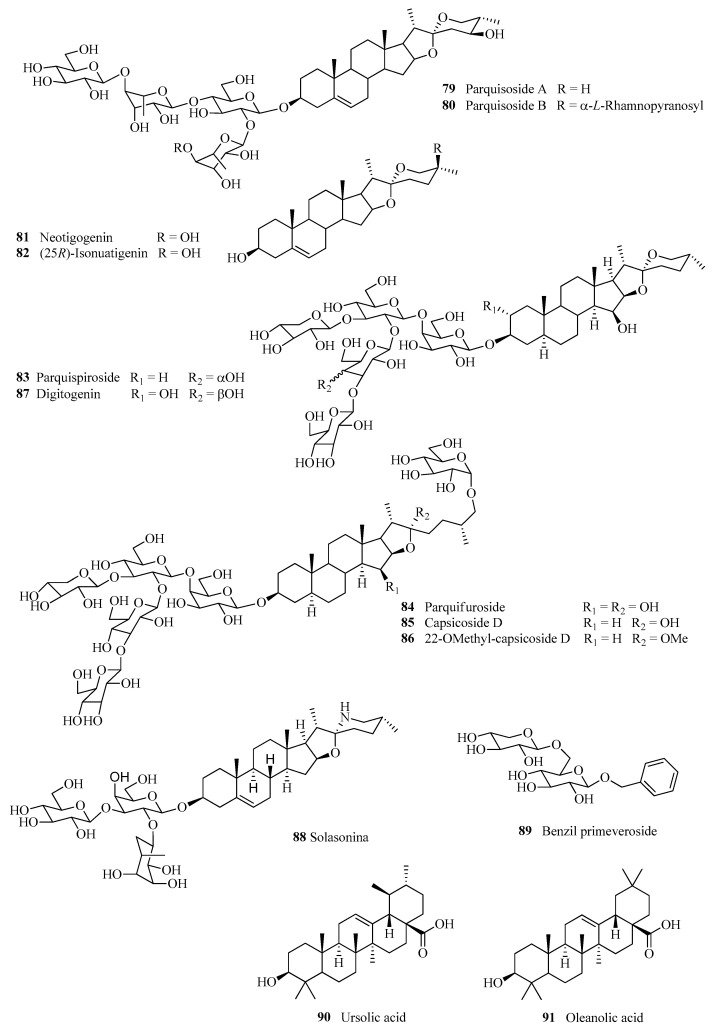
Saponins and aromatic glycosides.

The bark and, especially, the leaves of this shrub contain a large number of secondary metabolites, generally of low molecular weight. These metabolites have been isolated from polar infusions obtained via extraction with methanol and/or ethanol/water (Scheme 1), purified using direct and reverse-phase chromatographic techniques (on column or HPLC), and identified using spectroscopic techniques (NMR, both one-dimensional and two-dimensional) and mass spectrometry. Specifically, from the organic infusions, C_13_-nor-isoprenoids (**1**–**18**, Figure 3), sesquiterpenes (**19**–**20**, Figure 4), a spirostane (**21**, Figure 4), a pseudosapogenin (**22**, Figure 4), lignans (**23**–**42**, Figure 5), aromatic compounds (**43**–**47** and **89**, Figure 6), oxylipins (**63**–**76**, Figure 8), kaurenic glycosides (**77**–**78**, Figure 9), steroid saponins (**79**–**86**, Figure 10), an aromatic glycoside (**89**, Figure 10), and pentacyclic triterpenes (**90**–**91**, Figure 10) have been isolated. Conversely, from the aqueous infusion, aromatic compounds (**48**–**58**, Figure 6), flavones (**60**–**62**, Figure 7), a steroid saponin (**87**, Figure 10), and a glycoalkaloid (**88**, Figure 10) have been isolated.

### 3.4. Herbicidal Activity 

From the fresh leaves of *C. parqui*, a total of 76 compounds were isolated (Table 2), which after purification and structural determination were tested to evaluate their phytotoxic activity. In particular, the fresh leaves were finely chopped and then infused with methanol, methanol/water: 9/1 (*v*/*v*), water/ethanol: 1/1 (*v*/*v*) + 1% NH_4_OH, and water, respectively. The last three infusions were dried and then extracted with solvents of increasing polarity as indicated in Scheme 1. Thus, after numerous chromatographic steps, the following compounds were isolated: the C_13_-nor-isoprenoids [42,43]; the sesquiterpenes [43]; the spirostan [43]; the pseudosapogenin [43]; the lignans [44,45]; the aromatic compounds 43–59 [46,47]; the flavones [46]; and the oxylipins [48].

The compounds **1**–**76** were subjected to phytotoxicity assays to evaluate their effects on seed germination and the growth of roots and seedlings of various target organisms, including *Lactuca sativa*, *Solanum lycopersicum*, *Amaranthus retroflexus*, *Chenopodium album*, *Potamogeton oleracea*, and *Allium cepa* (Table 3). The assays were performed at different concentrations ranging from 10^−4^ to 10^−9^ M, following the protocol developed by Macias et al. [54], using the well-known herbicide Pendimethalin as a reference.

#### 3.4.1. Assay with C_13_-nor-Isoprenoids (**1**–**18**), Sesquiterpenes (**19**–**20**), Spirostane (**21**), and Pseudosapogenin (**22**)

Except for nor-terpenes **3**, **21**, and **22**, the tested compounds had no effect on germination but showed moderate inhibitory activity on root and shoot growth. The activity of the glycosylated compound **22** is intriguing, considering that glycosylation is the main detoxification mechanism adopted by plants to defend themselves against phytotoxic substances which they produce and store [55]. Among all the compounds tested, spirostane **21** was the most active, with root and shoot elongations reduced by up to 60% and germination by up to 30% at a concentration of 10^−4^ M. In general, the more polar compounds that are more soluble in water appear to be more active.

#### 3.4.2. Assay with Lignans (**23**–**35**, **40**–**42**)

Compounds **23**–**35** and **40**–**42** were tested on *A. retroflexus*, *P. oleracea*, and *C. album*, in a concentration range varying from 10^−4^ to 10^−8^ M. Lignans **23**–**26** were the most active on *A. retroflexus*, inhibiting its germination even at the lowest concentration, while compounds **29**–**30** showed anti-germination activity on *P. oleracea* and anti-radical activity on *A. retroflexus*. All compounds were slightly stimulating for shoot elongation of *C. album* and *P. oleracea*.

#### 3.4.3. Assay with Lignans (**36**–**39**)

These compounds were tested on *L. sativa* and *S. lycopersicum*, showing low phytotoxic activity on both. Of the four compounds in question, only compound **39** was able to inhibit the shoot length of *S. lycopersicum* by about 50% at a concentration of 10^−4^ M.

#### 3.4.4. Assay with Aromatic Compounds (**43**–**59**) and Flavones (**60**–**62**)

The aqueous infusion of *C. parqui* leaves was tested on the germination, root length, and shoot length of *L. sativa*, *S. lycopersicum*, and *A. cepa* [39]. The interesting results obtained suggested dividing the entire extract into three fractions, two obtained via extraction with methylene chloride and ethyl acetate, while the third was the remaining aqueous part (Scheme 1). From the first organic fraction, compounds **48**–**50**, **52**–**53**, **55**–**56**, **60**, and **62** were isolated, while from the second fraction, compounds **43**–**47**, **51**, **54**, **57**–**58**, and **61** were isolated. Compounds **43**–**62** were tested on the same target organisms used for the phytotoxic evaluation of the aqueous extract, and some of them were far more active than the herbicides used as reference standards.

Only aromatic compounds **55** and **56** on *L. sativa* and chalcone **60** on *A. cepa* showed weak inhibitory effects on germination, while all others were practically inactive. Results on root elongation showed that some compounds, such as product **45**, could have a phytotoxic effect on *S. lycopersicum* but have a stimulating effect on *A. cepa*, or compound **44**, stimulating for *A. cepa* but inhibiting for *L. sativa*. Compounds **45** and **48** were able to inhibit the shoot length of *S. lycopersicum* and *A. cepa* by 66% and 60%, respectively, at a concentration of 1 nM [46].

#### 3.4.5. Assay with Oxylipins (**63**–**76**)

The oxylipins were also tested on *L. sativa* seeds but in a narrower concentration range, specifically between 10^−4^ and 10^−8^ M [48]. It is not easy to rationalize the results of the phytotoxicity of these compounds. For example, at a concentration of 10^−4^ M, compounds **63**–**68** showed weak inhibitory action on germination, with values around 10%, and action on the elongation of the hypocotyl and root with inhibition values around 20%. However, at a concentration 100 times lower, only compounds **65** and **66** maintained weak inhibitory activity on root growth, while the corresponding alcohols **63** and **64**, or the corresponding methyl esters **67** and **68**, were even slightly stimulatory. Alternatively, compounds **69** and **71** stimulate germination and inhibit radical elongation, while compounds **70** and **72** inhibit germination and stimulate radical elongation. In general, it seems that the compounds present phytotoxicity values closely related to their degree of unsaturation, as for compounds **63**–**68**, **73**, and **74**, or phytotoxicity values dependent on the number of hydroxyl functions, as for compounds **75** and **76**.

It is interesting to note that oxylipins seem to play a crucial role in intra- and extracellular communication in vertebrates, fungi, and plants. In microorganisms, these metabolites are involved in the regulation of cell growth and differentiation, while in plants, their role in defense mechanisms based on apoptosis processes in response to infections caused by pathogens seems to be proven.

### 3.5. Other Isolated Metabolites 

#### 3.5.1. Kaurenic Glycosides (**77**–**78**) with Strychnine-Like Action

Two kaurenic glycosides named carbossiparquin (**77**) and parquin (**78**) have been isolated from the leaves of *C. parqui*, whose structures have been determined using NMR techniques and mass spectrometry [56] (Figure 9 and Table 4).

These compounds are structurally very similar, differing only in the presence of a second carboxylic function at carbon C-4 of the first compound. It is noteworthy that compounds **77** and **78** are quite like two toxins with strychnine-like action, namely carboxyatractyloside (A) and atractyloside (B), isolated from *Atractylis gummifera* [57]. In mice, carboxiparquin (**77**) has an LD_50_ value of 4.3 mg kg^−1^ and is over 50-times more toxic than crude extracts of *C. parqui* leaves. It is interesting to note that this toxin causes lesions in both the kidneys and the liver, like those observed in animals intoxicated after consuming *C. parqui*. The second compound (**78**) is relatively non-toxic and considered essentially a co-metabolite.

#### 3.5.2. Cytotoxic Secondary Metabolites 

Four new steroid saponins have also been isolated, three of which are monodesmosidic, called parquisoside A (**79**) and B (**80**) [58] and parquispiroside (**83**) [59], along with compound **84**, named parquifuroside [59]; together with the known steroid saponins: neotigogenin (**81**) [60] and (25R)-isonuatigenin (**82**) [61], capsicoside D (**85**) [62], 22-*O*-methyl-capsicoside D (**86**) [61], and digitogenin (**87**) [25,61]; the glycoalkaloid solasoninee (**88**) [62]; and the aromatic glycoside benzyl primeveroside (**89**) [59] (Figure 10 and Table 4). If compounds **79** and **80** are likely capable of inhibiting carrageenan-induced edema, there is no definitive evidence to support this. However, compounds **81**–**83** and **86**–**89** were tested for their cytotoxicity on four human cell lines: HeLa, HepG2, U87, and MCF7. Of these latter five compounds, only compound **81** showed moderate activity, with IC50 values of 7.7, 7.2, 14.1, and 3.3 μM, respectively. These values are quite promising considering that cisplatin, an antineoplastic chemotherapeutic agent used in the treatment of numerous tumors but with significant side effects, has much higher LC_50_ values of 39.2, 14.6, 7.3, and 23.0 μM, respectively [58].

**Table 4:** Other metabolites isolated from the leaves of *C. parqui*.

No.	Common Name/IUPAC Name	Ref.
**77**	Carboxiparquin	[56]
**78**	Parquin	
**79**	Parquisoside A/(3β,24S,25S)-spirost-5-ene-3,24-diol 3-O-{[α-L-rhamnopyranosyl-(1→2)]-β-D-glucopyranosyl-(1→4)-α-L-rhamnopyranosyl-(1→4)]-β-D-glucopyranoside	[58]
**80**	Parquisoside B/(3β,24S,25S)-spirost-5-ene-3,24-diol 3-O-{[α -L-rhamnopyranosyl-(1→4)-α-L-rhamnopyranosyl-(1→2)]-β-D-glucopyranosyl-(1→4)-α-L-rhamnopyranosyl-(1→4)}-β-D-glucopyranoside	[58]
**81**	Neotigogenin	[59]
**82**	(25R)-Isonuatigenin	[59]
**83**	Parquispiroside/25(R)-3β-[(O-β-D-glucopyranosyl-(1→3)-β-D-glucopyranosyl-(1→2)-O-[β-D-xylopyranosyl-(1→3)-O-β-D-glucopyranosyl-(1→4)-β-D-galactopyranosyl)oxy]-5α,15β,22R,25R-spirostan-3,15-diol	[58]
**84**	Parquifuroside/25(R)-26-[(β-D-Glucopyranosyl)oxy]-(3β[(O-β-D-glucopyranosyl-(1→3)-β-D-glucopyranosyl-(1→2)-O-[β-D-xylopyranosyl-(1→3)-O-β-D-glucopyranosyl-(1→4)-β-D-galactopyranosyl)oxy],5α,15β,22R,25R)-furostane-3,15,22-triol	[58]
**85**	Capsicoside D	[62]
**86**	22-O-Methylcapsicoside D	[58]
**87**	Digitogenin	[25,62]
**88**	Solasonine	[62]
**89**	Benzyl primeveroside	[58]
**90**	Ursolic acid	[58]
**91**	Oleanolic acid	[19,62]

## 4. Conclusions

Many Solanaceae are edible plants and are essential for human nutrition, but there are some which are extremely toxic, such as *Cestrum parqui* L’Herit, to the point that exposure to its leaves can cause respiratory difficulty, nausea, headache, and other unpleasant symptoms. 

In the traditional medicine of some countries, *C. parqui* is used as an antipyretic and for the treatment of fever and inflammation. Its extracts showed moderate antimicrobial activity and possible anticancer and antiproliferative action on specific cell lines. Numerous studies have allowed for the isolation and the structural determination of just under one hundred secondary metabolites, such as C_13_-nor-isoprenoids, sesquiterpenes, lignans, aromatic compounds, flavones, kaurenic glycosides, saponins, and alkaloids. Many of these compounds, but not all, have been studied to evaluate their insecticidal, antifeedant, and herbicidal activities both on weedy plants such as *A. retroflexus*, *P. oleracea*, and *C. album*, and on cultivated plants such as *A. cepa*, *L. sativa*, and *S. lycopersicum*.

In several cases, the activities measured proved to be higher. However, to date, the results are not definitive because it is not yet clear whether it is preferable to use an alcoholic or hydroalcoholic extract of the plant leaves or the individual metabolites. All of this suggests and justifies the significant interest in this plant, with possible and concrete commercial application.

## Figures and Tables

**Table 1:** Phylogenetic taxonomy of *C. parqui* [4,9,10].

Kingdom	Plantae
Sub-kingdom	Tracheophytes
Division	Angiosperms
Class	Eudicots
Sub-class	Asterids
Order	Lamianae Takht
Sub-order	Solanales Juss. ex Bercht. and J. Presl
Family	Solanaceae’ Juss
Tribe	Cestreae
Genus	*Cestrum* L.
Species	*parqui*

**Table 2:** Secondary metabolites isolated from the leaves of *C. parqui* and tested for their potential phytotoxic activity in different studies.

No.	Name	Ref
***C_13_-nor-isoprenoids***		
**1**	(6R,9R)-9-Hydroxy-4-megastigmen-3-one	
**2**	(2R,6R,9R)-2,9-Dihydroxy-4-megastigmen-3-one	
**3**	Byzantionoside A	
**4**	Annuionone E	
**5**	(3R,6R,7E,9R)-3,9-Dihydroxy-4,7-megastigmadiene	
**6**	(3R,6R,7E)-3-Hydroxy-4,7-megastigmadien-9-one	
**7**	(6R,7E,9R)-9-Hydroxy-4,7-megastigmadien-3-one	
**8**	Byzantionoside B	
**9**	Corcoionolo C	
**10**	(3S,5R,6R,7E,9R)-3,5,6,9-Tetrahydroxy-7-megastigmene	[42,46]
**11**	(3S,5R,6R,7E,9R)-5,6,9-Trihydroxy-3-isopropyloxy-7-megastigmene	
**12**	4-Oxo-β-ionol/(7E)-9-Hydroxy-5,7-megastigmadien-4-one	
**13**	(3S,7E,9R)-3,9-Dihydroxy-5,7-megastigmadiene	
**14**	(3R,7E)-3-Hydroxy-5,7-megastigmadien-9-one	
**15**	(3S,5R,6S,7E)-5,6-Epoxy-3-hydroxy-7-megastigmen-9-one	
**16**	(3S,5R,6S,7E,9R)-5,6-Epoxy-3,9-dihydroxy-7-megastigmene	
**17**	(6E,9S)-9-Hydroxy-4,6-megastigmadien-3-one	
**18**	(6Z,9S)-9-Hydroxy-4,6-megastigmadien-3-one	
***Sesquiterpenes***		
**19**	12-Hydroxy-α-cyperone	[43]
**20**	1,2,2a,3,6,7,8,8a-Octahydro-7-hydroxy-2a,7,8-trimethylacenaphthylen-4(4H)-one	[49,50]
***Spirostane***		
**21**	5α-Spirostan-3β,12β,15α-triol	[43]
***Pseudosapogenine***		
**22**	26-O-(30-Isopentanoyl)-β-D-glucopyranosyl-5α-furost-20(22)-ene-3β,26-diol	[43]
***Lignans Diepoxylignans***		
**23**	(+)-Pinoresinol	[44]
**24**	(+)-Mediaresinol	
**25**	(+)-Syringaresinol	
**26**	β-D-Glucopyranoside, 2,6-dimethoxy-4-[(1S,3aR,4S,6aR)-tetrahydro-4-(4-hydroxy-3,5-dimethoxyphenyl)-1H,3H-furo[3,4-c]furan-1-yl]phenyl	
***Epoxylignans***		
**27**	(+)-Lariciresinol	[44]
**28**	(+)-Justiciresinol	
**29**	5′-Methoxylariciresinol	
**30**	(−)-Berchemol	
***Neolignans***		
**31**	cis-Dehydrodiconiferyl alcohol	[44]
**32**	*trans*-Dehydrodiconiferyl alcohol	
**33**	(−)-Simulanol,	
***Sesquilignans***		
**34**	rel-(7Z,7′β,7″β,8′α,8″α)-4″,9,9′,9″-Tetrahydroxy-3,3′,3″-trimethoxy-4,7′:4′,7″-diepoxy-5,8′:5′,8″-sesquilign-7-ene	[44]
**35**	Herpetotriol	
**36**	rel-(7Z,7′α,7″α,8′β,8″β)-4″,9,9′,9″-Tetrahydroxy-3,3′,3″-trimethoxy-4,7′:4′,7″-diepoxy-5,8′:5′,8″-sesquilign-7-ene	
**37**	rel-(7E,7′α,7″α,8′β,8″β)-4″,9,9′,9″-Tetrahydroxy-3,3′,3″-trimethoxy-4,7′:4′,7″-diepoxy-5,8′:5′,8″-sesquilign-7-ene	
**40**	threo-4′,4″,7″,9″-Tetrahydroxy-3,3′,3″,5′-tetramethoxy- 4,8″-oxy-7,9′:7′,9-diepoxylignan	
**41**	erythro-4′,4″,7″,9″-Tetrahydroxy-3,3′,3″,5′-tetramethoxy- 4,8″-oxy-7,9′:7′,9-diepoxylignan	
**42**	erythro-4′,4″,7″,9″-Tetrahydroxy- 3,3′,3″,5,5′-pentamethoxy-4,8″-oxy-7,9′:7′,9-diepoxylignan	
***Oxyneolignan***		
**38**	Dimethyl (7′E)-3,3′-dimethoxy-4,40-oxyneolign-7′-ene-9,9′-dioate	[44,45]
***Norlignan***		
**39**	9′-Nor-3′,4,4′-trihydroxy-3,5-dimethoxylign-7-eno-9,7′-lactone	[44,45]
***Aromatic compounds***		
**43**	4-Hydroxybenzaldehyde	[46,47,51,52,53]
**44**	3,5-Dimethoxybenzaldehyde	
**45**	4-Hydroxybenzoic acid	
**46**	Vanillic acid	
**47**	Syringic acid	
**48**	Methyl 4-hydroxybenzoate	
**49**	Methyl vanillate	
**50**	Methyl syringate	
**51**	Tirosol	
**52**	3′,5′-Dimethoxy-4′-hydroxy-2-hydroxy-acetophenone	
**53**	p-Coumaric acid	
**54**	Caffeic acid	
**55**	Methyl ferulate	
**56**	Methyl ester of caffeic acid	
**57**	p-Dihydrocoumaric acid	
**58**	Methyl ester of p-dihydrocoumaric acid	
**59**	Dihydrosynapic acid	
***Flavones***		
**60**	4′-Hydroxy-4-methoxychalcon	[46]
**61**	Quercitin	
**62**	N-(p-Carboxymethylphenyl)-p-hydroxybenzamide	
***Oxylipins***		
**63**	(8S,9R,10E,12R,14Z)-Heptadeca-10,14-diene-1,8,9,12-tetraol	[48]
**64**	(8S,9R,10E,12S,14Z)-Heptadeca-10,14-diene-1,8,9,12-tetraol	
**65**	(9S,10R,11E,13R,15Z)-9,10,13-Trihydroxyoctadeca-11,15-dienoic acid	
**66**	(9S,10R,11E,13S,15Z)-9,10,13-Trihydroxyoctadeca-11,15-dienoic acid	
**67**	Methyl (9S,10R,11E,13R,15Z)-9,10,13-trihydroxyoctadeca-11,15-dienoate	
**68**	Methyl (9S,10R,11E,13S,15Z)-9,10,13-trihydroxyoctadeca-11,15-dienoate	
**69**	(9S,10R,11E,13R)-9,10,13-Trihydroxyoctadec-11-enoic acid	
**70**	(9S,10R,11E,13S)-9,10,13-Trihydroxyoctadec-11-enoic acid	
**71**	Methyl (9S,10R,11E,13R)-9,10,13-trihydroxyoctadec-11-enoate	
**72**	Methyl (9S,10R,11E,13S)-9,10,13-trihydroxyoctadec-11-enoate	
**73**	(8S,9S,10R,11Z,14Z)-Heptadeca-11,14-diene-1,8,9,10-tetraol	
**74**	(9S,10S,11R,12Z,15Z)-9,10,11-Trihydoxyoctadeca-12,15-dienoic acid	
**75**	Methyl 10-hydroxyoctadec-12-enoate	
**76**	Methyl (9S,10E,12Z,15Z)-octadeca-10,12,15-trien-9-ol	

**Table 3:** Range of tested concentrations and target organisms for the phytotoxicity tests of the compounds **1**–**76**.

Compounds	Range of Concentrations	Organism Test					
		*L. sativa*	*S.* *lycopersicum*	*A. retroflexus*	*C. album*	*P. oleracea*	*A. cepa*
**1**–**13**	10^−4^–10^−7^ M	x					
**14**–**18**	10^−5^–10^−7^ M	x					
**19**–**22**	10^−4^–10^−7^ M	x					
**23**–**35**	10^−4^–10^−8^ M			x	x	x	
**36**–**39**	10^−4^–10^−7^ M	x	x				
**40**–**42**	10^−4^–10^−8^ M			x	x	x	
**43**–**59**	10^−4^–10^−9^ M	x	x				x
**60**–**62**	10^−4^–10^−9^ M	x	x				x
**63**–**76**	10^−4^–10^−8^ M	x					

## Data Availability

Not applicable.

## References

[1] Dewick P.M. (2002). Medicinal Natural Products: A Biosynthetic Approach.

[2] Zhan C., Shen S., Yang C., Liu Z., Fernie A.R., Graham I.A., Luo J. (2022). Plant metabolic gene clusters in the multi-omics era. Trends Plant Sci..

[3] Souto A.L., Sylvestre M., Tölke E.D., Tavares J.F., Barbosa-Filho J.M., Cebrián-Torrejón G. (2021). Plant-Derived Pesticides as an Alternative to Pest Management and Sustainable Agricultural Production: Prospects, Applications and Challenges. Molecules.

[4] https://keyserver.lucidcentral.org/weeds/data/media/Html/cestrum_parqui.htm.

[5] L’Héritier de Brutelle C.L., Redouté P.J., Fréret L. (1784). Stirpes Novae aut Minus Cognitae, Quas Descriptionibus et Iconibus.

[6] Ruiz H. (1940). Travels of Ruiz, Pavon, and Dombey in Peru and Chile.

[7] Hunziker A.T. (2001). Genera Solanacearum: The Genera of the Solanaceae Illustrated, Arranged According to a New System.

[8] https://www.actaplantarum.org/flora/flora_info.php?id=2055&nnn=Cestrum%20parqui%20L%27H%C3%A9r.

[9] Olmstead R.G., Bohs L., Migid H.A., Santiago-Valentin E., Garcia V.F., Collier S.M. (2008). A molecular phylogeny of the Solanaceae. Taxon.

[10] Olmstead R.G. (2013). Phylogeny and Biogeography in Solanaceae, Verbenaceae and Bignoniaceae: A Comparison of Continental and Intercontinental Diversification Patterns. Bot. J. Linn. Soc..

[11] https://powo.science.kew.org/taxon/urn:lsid:ipni.org:names:30046363-2.

[12] https://europlusmed.org/cdm_dataportal/taxon/51664b74-b63f-4381-af29-46de5ef8d171.

[13] www.worldplants.de.

[14] Groves R.H., Boden R., Lonsdale W.M. (2005). Jumping the Garden Fence: Invasive Garden Plants in Australia and Their Environmental and Agricultural Impacts.

[15] Estomba D., Ladio A., Lozada M. (2005). Plantas medicinales utilizadas por una comunidad Mapuche en las cercanías de Junín de los Andes, Neuquén. BLACPMA.

[16] Backhouse N., Delporte C., Negrete R., Salinas P., Pinto A., Aravena S., Cassels B.K. (1996). Antiinflammatory and Antipyretic Activities of Cuscuta chilensis, *Cestrum parqui*, and Psoralea glandulosa. Int. J. Pharmacogn. Pharm..

[17] Molgaard P., Holler J.G., Asar B., Liberna I., Rosenbæk L.B., Jebjerg C.P., Jørgensen L., Lauritzen J., Guzman A., Adsersen A. (2011). Antimicrobial evaluation of Huilliche plant medicine used to treat wounds. J. Ethnopharmacol..

[18] Chenni H. (2015). Apoptosis induction by *Cestrum parqui* L’Hér. leaves on HL-60 Cell Line: Identification of active phytomolecules. Int. J. Cancer. Stud. Res..

[19] Chenni H. (2015). Identification of esterified oleanolic acid in *Cestrum parqui* leaves and its apoptotic induction on HT-29 cell line. J. Med. Pharm. Innov..

[20] Falkenberg S.S., Tarnow I., Guzman A., Mølgaard P., Simonsen H.T. (2012). Mapuche herbal medicine inhibits blood platelet aggregation. Evid. Based Complement. Alternat. Med..

[21] Souad K., Ali S., Mounir A., Mounir T.M. (2007). Spermicidal activity of extract from *Cestrum parqui*. Contraception.

[22] El-Sayed A.S.A., George N.M., Abou-Elnour A., El-Mekkawy R.M., El-Demerdash M.M. (2023). Production and bioprocessing of camptothecin from *Aspergillus terreus*, an endophyte of *Cestrum parqui*, restoring their biosynthetic potency by *Citrus limonum* peel extracts. Microb. Cell Fact..

[23] Demain A.L., Vaishnav P. (2011). Natural products for cancer chemotherapy. Microb. Biotechnol..

[24] Canham P.A.S., Warren F.L. (1950). Saponins II. Isolation of gitogenin and digitogenin from *Cestrum parqui*. S. Afr. J. Chem..

[25] Bianchi E., Girardi F., Diaz F., Sandoval R., Gonzales M. (1963). Components of the leaves and fruit of *Cestrum parqui*: Tigogenin, digallogenin, digitogenin and ursolic acid. Ann. Chim.-Rom..

[26] Bezerra J.J.L., Pinheiro A.A.V., de Lucena R.B. (2023). Poisoning in ruminants caused by species of the genus *Cestrum* L. (Solanaceae) in Brazil: A review of toxicological and phytochemical evidence. Toxicon.

[27] Brevis C., Quezada M., Sierra M.A., Carrasco L., Ruiz A. (1999). Lesiones observadas en intoxicaciones accidentales con *Cestrum parqui* (L’Herit) en bovinos. Arch.Med. Vet..

[28] Barbouche N., Hajem B., Lognay G., Ammar M. (2001). Contribution for studying the biological activity of the leaves extracts of *Cestrum parqui* L’Herit. on desert locust *Schistocera gregaria*. Biotechnol. Agron. Soc..

[29] Ikbal C., Ben H.K., Ben H.M. (2006). Insect growth regulator activity of *Cestrum parqui* saponins: An interaction with cholesterol metabolism. Commun. Agric. Appl. Biol. Sci..

[30] Zapata N., Budia F., Viñuela E., Medina P. (2006). Insecticidal effects of various concentrations of selected extractions of *Cestrum parqui* on adult and immature Ceratitis capitata. J. Econ. Entomol..

[31] Thomas M.C., Heppner J.B., Woodruff R.E., Weems H.V., Steck G.J., Fasulo T.R. (2001). Mediterranean Fruit Fly, Ceratitis Capitata. (Wiedemann) (Insecta: Diptera, Tephritidae).

[32] Huerta A., Chiffelle I., Araya L., Curkovic T., Araya J.E. (2021). Insecticidal capacity of *Cestrum parqui* (Solanaceae) leaf extracts on *Xanthogaleruca luteola* (Coleoptera: Chrysomelidae) adults. Rev. Colomb. Entomol..

[33] Ben Hamouda A., Ammar M., Habib M., Hamouda B. (2011). Effect of Olea europea and *Cestrum parquii* Leaves on the Cuticle and Brain of the Desert Locust, *Schistocerca gregaria* Forsk (Orthoptera:Acrididae). Pest Technol..

[34] Chaieb I., Halima-Kamel M.B., Ben M.H. (2009). Antifeedant activity of *Cestrum parqui* crude saponic extract. Tunis. J. Med. Plants Nat. Prod..

[35] Chaieb I., Mounia B.H., Habib B.H. (2007). Toxicity experiments of the saponic extract of *Cestrum parqui* (Solanaceae) on some insect spices. J. Entomol..

[36] Chaieb I., Mounia B.H. (2001). The effect of food containing *Cestrum paquii* (Solanaceae) extract on various damaging Lepidoptera. Meded. Rijksuniv. Gent. Fak. Landbouwkd. Toegepaste Biol. Wet..

[37] Chaieb I., Habib B., Hichem B.J., Monia B.H., Habib B.H., Zine M. (2007). Purification of a natural insecticidal substance from *Cestrum parqui* (Solanaceae). PJBS.

[38] Chaieb I., Ben H., Trabelsi M., Hlawa W., Raouani N., Ben A., Daami M., Ben H. (2003). Pesticidal Potentialities of *Cestrum parqui* saponins. Int. J. Agric. Res..

[39] Huanquilef C., Espinoza J., Mutis A., Bardehle L., Hormazábal E., Urzúa A., Quiroz A. (2021). Antifeedant activities of organic fractions from *Cestrum parqui* leaves on the red-haired bark beetle *Hylurgus ligniperda*. J. Soil Sci. Plant Nutrit..

[40] Chaieb I., Halima-Kamel M.B., Hammouda M.B. (2005). Experiments for studying the molluscicidal potential of *Cestrum parqui* (Solanaceae) saponins against *Theba pisana* (Helicidae) snails. Comm. Agric. Appl. Biol. Sci..

[41] Chaieb I., Tayeb W. (2009). Comparison of the molluscicidal activity of *Cestrum parqui* (Solanaceae) and *Quillaja saponaria* (Quillajaceae) saponins. Tunis. J. Med. Plants Nat. Prod..

[42] D’Abrosca B., DellaGreca M., Fiorentino A., Monaco P., Oriano P., Temussi F. (2004). Structure elucidation and phytotoxicity of C_13_-nor-isoprenoids from *Cestrum parqui*. Phytochemistry.

[43] D’Abrosca B., DellaGreca M., Fiorentino A., Monaco P., Natale A., Oriano P., Zarrelli A. (2005). Structural characterization of phytotoxic terpenoids from *Cestrum parqui*. Phytochemistry.

[44] Fiorentino A., DellaGreca M., D’Abrosca B., Oriano P., Golino A., Izzo A., Monaco P. (2007). Lignans, neolignans and sesquilignans from *Cestrum parqui* l’Her. Biochem. Syst. Ecol..

[45] D’Abrosca B., DellaGreca M., Fiorentino A., Golino A., Monaco P., Zarrelli A. (2006). Isolation and characterization of new lignans from the leaves of *Cestrum parqui*. Nat. Prod. Res..

[46] D’Abrosca B., DellaGreca M., Fiorentino A., Monaco P., Zarrelli A. (2004). Low molecular weight phenols from the bioactive aqueous fraction of *Cestrum parqui*. J. Agric. Food Chem..

[47] DellaGreca M., Fiorentino A., Izzo A., Napoli F., Purcaro R., Zarrelli A. (2007). Phytotoxicity of secondary metabolites from *Aptenia cordifolia*. Chem. Biodivers..

[48] Fiorentino A., D’Abrosca B., DellaGreca M., Izzo A., Natale A., Pascarella M.T., Pacifico S., Zarrelli A., Monaco P. (2008). Chemical characterization of new oxylipins from *Cestrum parqui*, and their effects on seed germination and early seedling growth. Chem. Biodivers..

[49] Cangiano T., DellaGreca M., Fiorentino A., Isidori M., Monaco P., Zarrelli A. (2001). Lactone diterpenes from the aquatic plant *Potamogeton natans*. Phytochemistry.

[50] Cangiano T., DellaGreca M., Fiorentino A., Isidori M., Monaco P. (2002). Effect of ent-labdane diterpenes from Potamogetonaceae on *Selenastrum capricornutum* and other aquatic organisms. J. Chem. Ecol..

[51] Pollio A., Romanucci V., Di Mauro A., Barra F., Pinto G., Crescenzi E., Roscetto E., Palumbo G. (2016). Polyphenolic profile and targeted bioactivity of methanolic extracts from Mediterranean ethnomedicinal plants on human cancer cell lines. Molecules.

[52] Cutillo F., D’Abrosca B., DellaGreca M., Zarrelli A. (2004). Chenoalbicin, a novel cinnamic acid amide alkaloid from *Chenopodium album*. Chem. Biodivers..

[53] DellaGreca M., Previtera L., Purcaro R., Zarrelli A. (2007). Cinnamic ester derivatives from Oxalis pes-caprae (*Bermuda buttercup*). J. Nat. Prod..

[54] Macıas F.A., Castellano D., Molinillo J.M.G. (2000). Search for standard phytotoxicity bioassay for allelochemicals. Selection of standard target species. J. Agric. Food Chem..

[55] le Roy J., Huss B., Creach A., Hawkins S., Neutelings G. (2016). Glycosylation is a major regulator of phenylpropanoid availability and biological activity in plants. Front. Plant Sci..

[56] Pearce C.M., Skelton N.J., Naylor S., Kanaan R., Kelland J., Oelrichs P.B., Sanders J.K.M., Williams D.H. (1992). Parquin and carboxyparquin, toxic kaurene glycosides from the shrub *Cestrum parqui*. J. Chem. Soc..

[57] Daniele C., Dahamna S., Firuzi O., Sekfali N., Saso L., Mazzanti G. (2005). *Atractylis gummifera* L. poisoning: An ethnopharmacological review. J. Ethnopharmacol..

[58] Baqai F.T., Ali A., Ahmad V. (2001). Two new spirostanol glycosides from *Cestrum parqui*. Helv. Chim. Acta.

[59] Mosad R.R., Ali M.H., Ibrahim M.T., Shaaban H.M., Emara M., Wahba A.E. (2017). New cytotoxic steroidal saponins from *Cestrum parqui*. Phytochem. Lett..

[60] Abdel-Gwad M.M., El-Amin S.M., El-Sayed M.M., Refahy L.A., Sabry W.A. (1997). Molluscicidal saponins from *Cestrum parqui*. AJPS.

[61] Torres R., Modak B., Faini F. (1988). (25R)-Isonuatigenin, an unusual steroidal sapogenin as taxonomicmarker in *Cestrum parqui* and Vestia lycioides. Biol. Soc. Chil. Quim..

[62] Silva M., Mancinelli P., Cheul M. (1962). Chemical study of *Cestrum parqui*. J. Pharm. Sci..

